# Evaluation of anticandidal activities and phytochemical examination of extracts prepared from *Vitex agnus-castus*: a possible alternative in treating candidiasis infections

**DOI:** 10.1186/s12906-022-03552-x

**Published:** 2022-03-15

**Authors:** Fatima O. Al-Otibi, Ghaida I. Alrumaizan, Raedah I. Alharbi

**Affiliations:** Botany and Microbiology Department, College of Science, King Saud University, P. O Box 22452, Riyadh, 11495 Saudi Arabia

**Keywords:** *Vitex agnus-castus*, *Candida* sp, Anticandidal activities, Phytochemical analysis, DPPH

## Abstract

**Background:**

Candidiasis infection is associated with high morbidity and mortality. Fungicidal resistance of some commercially used fungicides ascended the need for a naturally effective alternative. The current study aimed to evaluate the fungicidal activity of *Vitex agnus-castus* extracts in vitro against some *Candida* species.

**Methods:**

The bioactive compounds contained in the crude and alcoholic extracts were compared, and the antioxidant activity was tested, as well. The phytochemical analysis was carried out by Fourier Transform-Infrared Spectroscopy (FTIR) and Gas Chromatography-Mass Spectrometry (GC/MS). The scavenger activity of the tested extracts was tested, as well. The anticandidal activity was tested to detect the effect of the tested extracts on the mycelial growth of *Candida albicans, Candida krusei, Candida parapsilosis, Candia tropicalis, Candida famata, Candida rhodotorula, *and *Candida dublinesis*. The minimum inhibitory concentrations were calculated for all reported activities. The Scanning Electron Microscopy (SEM) and the Transmission Electron Microscopy (TEM) were used to detect the morphological and ultrastructure response in some selected species.

**Results:**

FTIR and GC/MS revealed the existence of different bioactive chemical groups such as polyphenols, fatty acids, terpenes, terpenoids, steroids, aldehydes, alcohols, and esters, phytol which is a diterpene. DPPH results confirmed the antioxidant activity of all extracts where the methanolic extract was the strongest scavenging substrate. All extracts showed strong inhibitory effects against different species at a concentration of 200 µg/ml (*P* < *0.001*). SEM and TEM showed morphological and ultrastructure changes in *C. famata.*

**Conclusion:**

The current study suggested a reliable antifungal activity of different extracts of *Vitex agnus-castus* against different *Candida* species and strains. However, further studies are required to confirm the safety of these extracts to be used in medical applications.

## Background

For almost 5,000 years, plants were used for food, beverage preparation, and medicinal purposes [1]. Medicinal plants possess therapeutic properties or exert beneficial pharmacological effects on the human body [2]. In the Arab world, in the Middle East (excluding Iran and Turkey) and North Africa (MENA) region, medicinal plants are interesting healthcare resources and salient elements of prophetic and folk medicine [3]. Scientific studies have proven that some plants, such as garlic, pomegranate*, *black seeds*, Costus, Miswak, Henna, ginger,* and Fenugreek are effective for treating human diseases [4]. Medicinal plants enrich the sources of components that can be used in drug/ therapeutic development [2].

Medicinal plants naturally synthesize and accumulate other secondary metabolites, like alkaloids, sterols, terpenes, flavonoids, etc. [5]. Secondary plant metabolites are numerous chemical compounds produced by the plant cell through metabolic pathways derived from the primary metabolic pathways [5]. These metabolites are referred to as active substances, which have beneficial physiological effects on living organisms [6]. They possess various biological effects, which provide the scientific base of herbs in traditional medicine in many ancient communities [7]. They have been described as antibiotics, anti-fungal, and antiviral agents, and, therefore, they can protect against different pathogens [8].

*Candidiasis* is a common infection caused by *Candida* sp., widespread in the environment [9]. A characteristic phenomenon of some *Candida* Species is their ability to commensal with other native species of bacteria to induce harmful colonization in different organs of the human body such as the mouth, gastrointestinal tract, and vagina [10]. In one of the most frequent fungal infections, urinary tract infections (UTI), *C. albicans* is considered the most common source of infection, with *C glabrata* and *C tropicalis *[11]*.* Despite the immune response initiated to control and resist the harmful candida infection, it was reported that the excessive usage of antibiotics, which might induce morphological and genetic changes in the native bacteria increase the acidity and chemical composition of the surrounding environment and permit the candida infection [12]. The resistance to the common anti-candida treatments is a well-known issue that was reported by different medical agencies and clinical studies [13, 14]. As been reported by the Centers for Disease Control and Prevention (CDC), more than 7% of all *Candida* infections, particularly by *C. albicans, C. parapsilosis,* *C. auris,* and *C. glabrata* were resistant to fluconazole [15]*.* That suggested searching for other possible anti-candida treatments such as natural plant extracts.

*Vitex* is a member of the family *Verbenaceae* and includes about 250 species distributed, worldwide [16]. *V. agnus-castus*, commonly known as chaste tree or sage tree, is a little deciduous tree or large shrub with a showy summertime flower display [17]. *V. agnus-castus* fruit is commonly traditionally employed for a range of female reproductive disorders, including premenstrual syndrome (PMS) and female hormonal imbalances such as the depression, cramps, mood swings, water retention, and weight gain associated with PMS, premenstrual dysphoric disorder (PMDD), lactation difficulties, low fertility, and menopause-related complaints [18]. Several phytochemical studies showed that different parts of *V. agnus-castus* (fruits, leaves, and flowering stems) contain numerous secondary metabolites, such as iridoids, flavonoids, terpenoids, essential oils, ketosteroids, and vanillic acid [19]. These bioactive components are naturally occurring in most plant materials, which are known to possess interesting biological properties such as antioxidant, anti-carcinogenic, antiviral, antibacterial, anti-diabetic, and anti-inflammatory activities [20, 21]. Despite that, *V. agnus-castus* is poorly studied for its clinical uses.

The current study aimed to test the anticandidal effect of different extracts of *V. agnus-castus* leaf against different *Candida* species assess their possible inhibitory effects.

## Materials and methods

### Plant material and preparation of extracts

A new fresh, disease-free, *Vitex agnus-castus* plant was purchased from a local herbal market in Al Haier region, Riyadh, Saudi Arabia**.** Plants were identified, authenticated, and classified by Dr. *Najat Bukhari*, a taxonomist from the Department of Botany and Microbiology, King Saud University, Riyadh, Saudi Arabia.

For the preparation of different extracts of *Vitex agnus-castus*, leaves were washed several times with tap water, distilled water, and dried at room temperature. An amount of 45 g of dried leaves was crushed and grounded in 450 ml of either 96% Ethanol, absolute Methanol, or sterile distilled water to prepare the ethanolic, methanolic, or aqueous extracts, respectively. The extracts were placed in a shaker at 145 rpm for 48 h and then filtered using gauze. The filtrates were evaporated by aeration at room temperature. The dried crude extracts were rediscovered in the corresponding solvent (ethanol, methanol, or dis, water) to prepare different concentrations (25, 50, 100, 200 µg/ml). Dimethyl Sulfoxide (DMSO) was used to enhance dissolving the extract [22].

### *Candida* strains

Eleven Candida strains were used to assess the antifungal activity of *Vitex agnus-castus*. The species were four strains purchased from (ATCC, Manassas, VA, United States) included *C. albicans* (ATCC® 60,193™), *C. krusei* (ATCC® 14,243™) (Ck1), *C. parapsilosis* (ATCC® 22,019™) (Cp1), *C. tropicalis* (ATCC® 66,029™). Other seven strains were obtained from either King Khalid University Hospital (KKUH), or King Abdullah International Medical Research Center (KAIMRC), Riyadh, Saudi Arabia, and included *C. parapsilosis* (Cp2)*, Candida famata, C. rhodotorula, C. dublinesis, C. auris, C. krusei* (Ck2), *and C. krusei* (Ck3). The fresh inoculum was prepared by subculturing the studied species onto a Sabouraud dextrose agar (SDA) medium at 28 °C for 48 h, as previously described [23]. The turbidity of growing *Candida* suspension was adjusted to match the turbidity standard of 0.5 McFarland units, by spectrophotometry tune of 0.1 OD and was read at 600 nm wavelength.

### Phytochemical analysis

Plant samples were analyzed as been described before [24].

#### Fourier transform infra-red spectroscopy (FT-IR) analysis

The dried powder of the aqueous extract of *V. agnus-castus* was used for FTIR analysis. Briefly, 10 mg of the dried extract powder was encapsulated in 100 mg of KBr pellet to prepare translucent sample discs. The powdered sample of each plant specimen was loaded in FTIR spectroscope FT-IR (Nicolet 6700 FT-IR Spectrometer, Waltham, MA, USA) [25].

#### Gas chromatography-mass spectrometry (GC–MS) analysis

The methanolic and ethanolic extracts were quantified by a gas chromatograph (Agilent Technologies, 7890A GC System). It was equipped with a column Agilent DB-WAX (30 m × 320 μm × 0.25 μm), coupled to a mass spectrometer Agilent Technologies (5975C V2-MSD with Triple-Axis Detector), with Autosampler and injector (G4513A) (Agilent Technologies, Santa Clara, CA, United States). The machine was programmed at temperature 60 °C for 5 min, then at 11 °C/min increments up to 250 °C. The injector flow rate was 250 °C; carrier gas was He of 99.9995% purity, column flow rate 1.2926 mL/min [25].

#### Determination of DPPH radical scavenging activity

A working solution of 2,2-diphenyl-1-picrylhydrazyl (DPPH) reagent at a concentration of 100 µM, was prepared as follows; by adding 4 mg of DPPH to 10 ml methanol and mixing well (Stock solution (SS) of 1 mM) and kept in dark. The methanol was diluted (1:10) and kept in darkness. A serial dilution of Ascorbic Acid (as a positive control) or tested reagents was prepared, by dilution in water and mix well (SS). The working solution (WS) (100 µg/ml) was prepared by diluting in water (1:100). In 96 well plates add the reagents (of ascorbic acid or tested reagents). Triplicates of each reagent were prepared and incubated in dark for 30 min. The absorbance was read at 517 nm [26]. The percentage inhibition of DPPH free radical scavenging activity was calculated as follows:

$$\mathrm{\% Inhibition}=\frac{Ac-At}{Ac}\times 100$$

where:

Ac = Absorbance of DPPH (concentration 0 µg/ml).

At = Absorbance of sample (extract/ascorbic acid).

#### Anti-*Candida* bioassay and determination of minimum inhibitory concentration (MIC)

The anticandidal activity of different extracts was evaluated by the “Well Diffusion Method” [27]. Briefly, 10 mL of SDA medium was poured into the sterile Petri dishes as a basal layer, followed by the addition of 15 mL seeded medium, previously inoculated with the prepared microbial suspension at the concentration of 1 mL of fungal suspension/100 mL medium, to attain a viable cell count of 10^5^ CFU/ml. The plates were further incubated at 35 °C for 48 h, where the inhibition zone diameter was measured using a Vernier caliper as an indication of antifungal activity. Terbinafine was used as a positive control at a concentration of 50 μg/disk. Interpretation criteria of terbinafine as the antifungal agent corresponding to the inhibition zone diameter was ≥ 20 mm.

MIC was defined as the lowest concentration of plant extract exhibiting antifungal activity. MIC was evaluated for all extracts of *V. agnus-castus, *as previously described [28]. MIC was calculated according to the following equation:

$$MIC=\frac{Lowest Conc. inhibit the growth+Highest conc. allow the growth }{2}$$

### Detection of the morphological and ultrastructure characterizations of Candida species

Morphological and ultrastructure characterizations of selected fungal growth as a response of plant extracts detected by Scanning electron microscopy and Transmission electron microscopy.

#### Scanning Electron Microscope (SEM)

Primary fixation by buffered Glutaraldehyde (2.5%) overnight performed in a refrigerator, then washed by phosphate buffer (pH 7.2) and later fixed by buffered Osmium Tetroxide 1%. Then, samples were dehydrated by a series of ethanol concentrations. It then freezes dried in a critical point dryer and mounted on gold plated stubs. Samples were observed through scanning under a JEOL scanning electron microscope. The treated samples were coated with gold in a vacuum evaporator and examined with a scanning electron microscope (SEM Quanta-250, FEI, Czech Republic) [29].

#### Transmission electron microscopy (TEM)

Primary fixation by buffered Glutaraldehyde (2.5%) overnight performed in a refrigerator, then washed by phosphate buffer (pH 7.2) followed by secondary fixation using buffered Osmium Tetroxide 1% overnight in a 4 °C. The sample was then dehydrated by a series of ethanol concentrations and embedded by a resin mixture of SPI-Pon™- Araldite® Epoxy Embedding Kit (SPI Supplies, West Chester, PA, United States). That was followed by cutting using a Leica UC6 ultramicrotome produced section thicknesses between 70–80 nm and then loaded on a copper grid. The samples were then stained by aqua's uranyl acetate and lead citrate and examined under Jeol JSM-1011 electron microscope (Akishima, Tokyo, Japan) [29].

### Statistical analysis

Means and standard deviations were calculated using IBM SPSS Statistics 22.0 (Armonk, NY, United States) [25].

## Results and discussion

### Phytochemical analysis of different extracts from V. agnus-castus revealed robust chemical composition

The widespread of pathogenic bacterial and fungal microbes is considered one of the problems, that threaten human existence. Several studies reported the continuous multidrug resistance, particularly, among *Candida* spp., which increased the efforts towards suitable substitutions. One of these efforts is the use of pump inhibitors associating drugs, such as Quinoxaline derivatives, which inhibit the activity of ATP-binding cassette (ABC) transporters, and further affected the mycelial growth of *C. albicans*, *C. parapsilosis*, *C. tropicalis*, *C. glabrata*, and *C. krusei* [30]. Another study showed that natural and synthetic treatment contained an adequate amount of quinolines, sulfur-containing heterocycles indoles, phenols, and pyridines can act as NorA efflux pump inhibitors, which can reduce the growth of *Staphylococcus aureus* [31]. In the current study, the phytochemical analysis of different extracts of *V. agnus-castus* showed the existence of variable phenolic compounds, which might act as efflux pump inhibitors and reduce antimicrobial resistance.

**Fig. 1:**
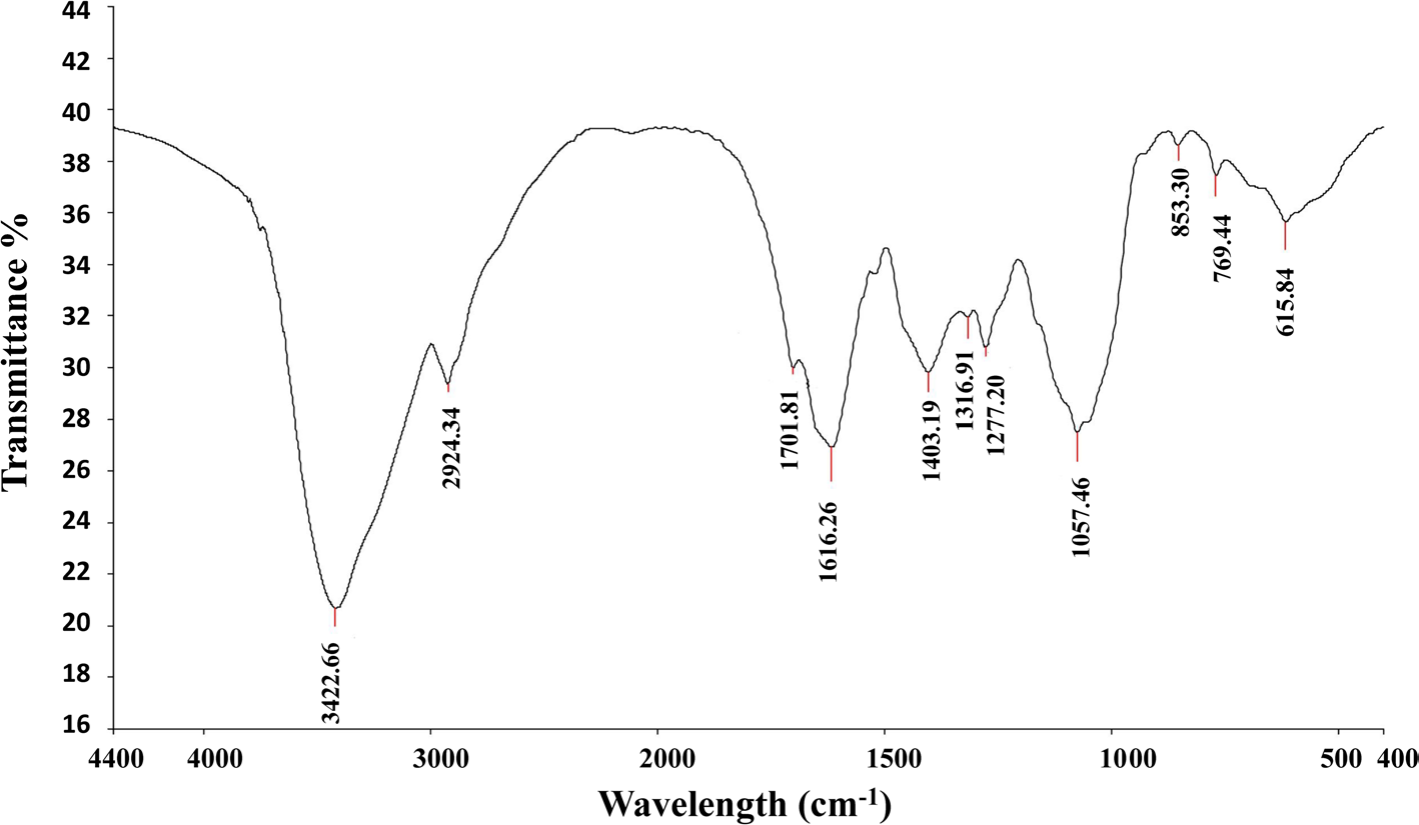
FT-IR results of the aqueous extract of *Vitex agnus-castus* L leaves. The results were produced by Nicolet 6700 FT-IR Spectrometer at the range of 500–4000/cm

The aqueous extract of *V. agnus-castus* was analyzed by FTIR to investigate the functional groups (Fig. 1). The results revealed the presence of OH-stretching associated with alcohols, phenols, or polyphenols. Furthermore, the peaks revealed the existence of several functional groups Hydroxyl, aromatic compounds, amine salts, and unsaturated ketones (Table 1). In agreement with our results, a previous study showed that the FTIR analysis of *V. agnus-castus* proved the presence of Alkenes, alkyl halides, Aromatic compounds, phenols, and hydroxyl functional groups [32]. Another study used FTIR in the analysis of *Amellia sinensis, Viola odorata, Commiphora mukul, V. agnus-castus, which* revealed the presence of (C = C) stretching for a peak at 1629 and 1631 cm-1, -COOH in the range 4000–1000 cm-1, C-O and OH-stretching (carboxylic acid groups) at 1384 and 3434 cm-1, CH2-stretching at 2850 and 2922 cm-1 [33].

**Table 1:** FT-IR analysis results

Absorption (cm-1)	Appearance	Group	Compound Class
3422	Strong, Broad	O–H Stretching	Alcohol
2924	strong, Broad	N–H Stretching	Amine Salt
1701	Strong	C = O Stretching	Conjugated aldehyde
1616	Strong	C = C Stretching	α, β-unsaturated ketone
1403	Strong	S = O stretching	Sulfate, Sulfonyl chloride
1316	Strong	C-N stretching	Aromatic amine
1277	Strong	C-O stretching	Aromatic ester
1057	Strong	C-O stretching	Primary alcohol
853, 769, 615	Strong	C–Cl stretching	Halo compounds

**Fig. 2:**
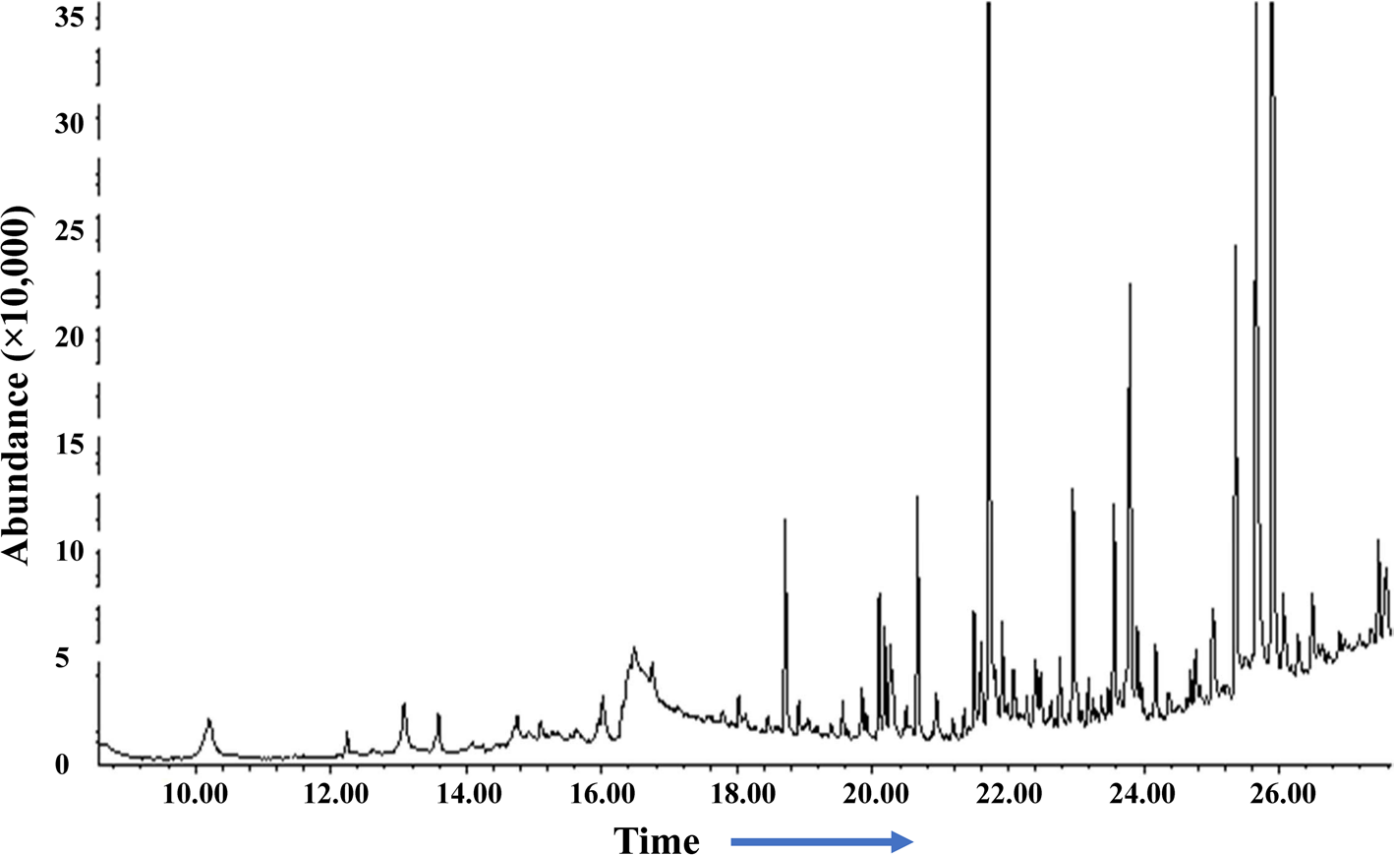
GC–MS chromatogram of *Vitex agnus-castus* leaves methanolic extract

**Fig. 3:**
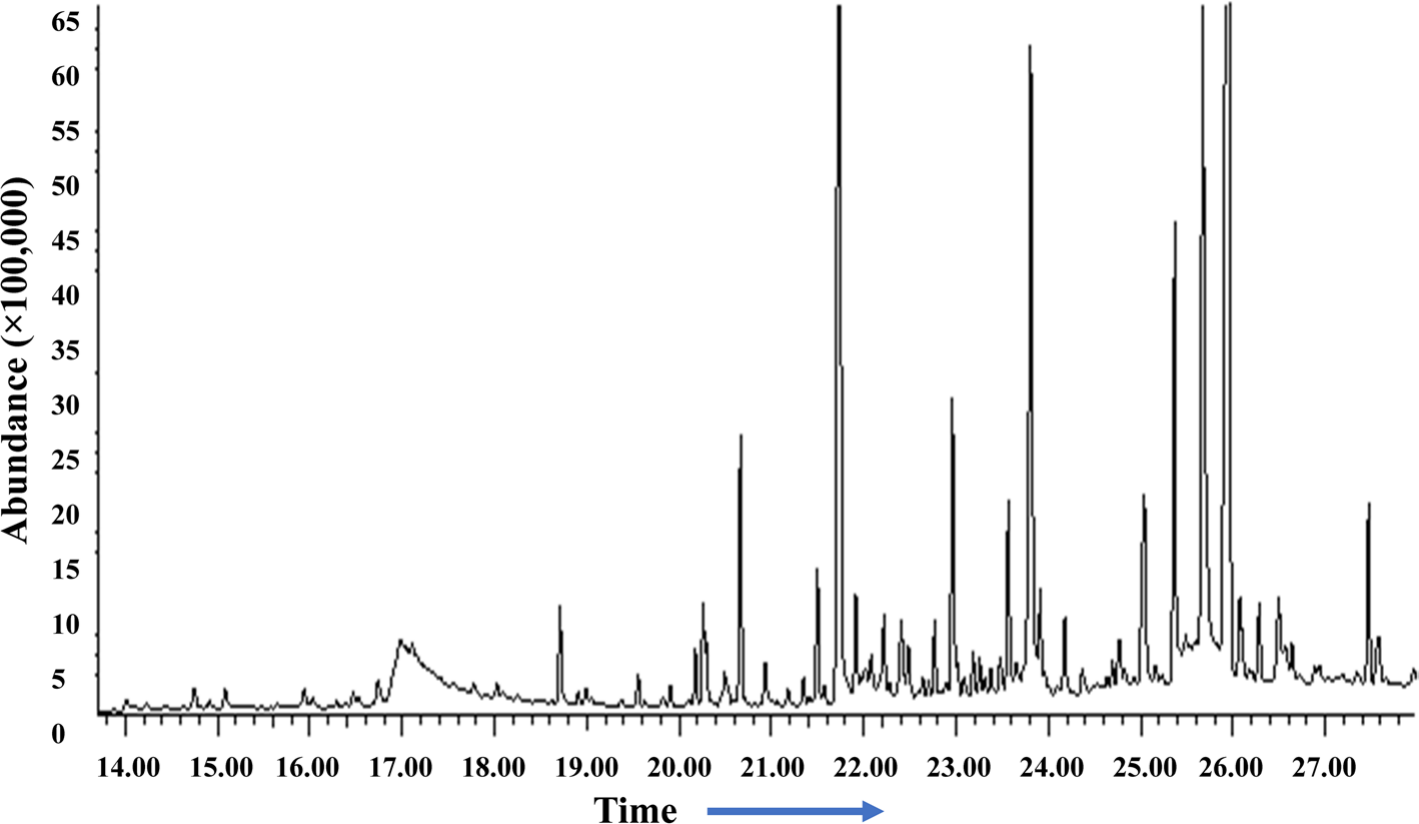
GC–MS chromatogram of *Vitex agnus-castus* leaves ethanolic extract

The results of GC/MS revealed that *V. agnus-castus* leaves extracts are rich in phytochemical components. A total of 34 chemical compounds were analyzed depending on the solvent type (Figs. 2 and 3). Ten compounds were investigated in the ethanolic extract (Table 2), twenty-two by methanolic extract (Table 3), and two compounds were shared in both solvents. These phytochemical compounds belong to different bioactive chemical groups such as polyphenols, fatty acids, terpenes, terpenoids, which have anticandidal activity, antioxidant, anti-inflammatory, and anticancer agents [34]. A previous study showed that plant extracts, in which Terbenoids were reported in their chemical composition, were suggested to have significant anticandidal activity [35]. Furthermore, steroids, flavonoids, phenolic acids, aldehydes, alcohols, esters, and phytol compounds were reported to have significant antiviral and antimicrobial activities [36]. In agreement with our results, a previous study showed that the extracts of *V. agnus castus* fruits and stems had more than 25 phenolic compounds with higher antioxidant activity such as luteolin, 3,4-dihydroxybenzoic acid, vanillic acid, chlorogenic acid, hesperidin [37]. In contrast to our findings, a previous study conducted the GC/MS analysis of *V. agnus castus* leaf extracts and revealed the existence of different chemical compositions included, 4,5-Dichloro-1,3-dioxolan-2-one, 1H-Indene, 2,3-dihydro-1,1,2,3,3-pentamethyl, and Isobutyl 4-hydroxybenzoate [38]. All these compounds are derivatives and constituents of different bioactive materials from middle eastern plants such as terpenes, fatty acids, steroids, polyphenols, which are known for their antimicrobial activity [39].

**Table 2:**
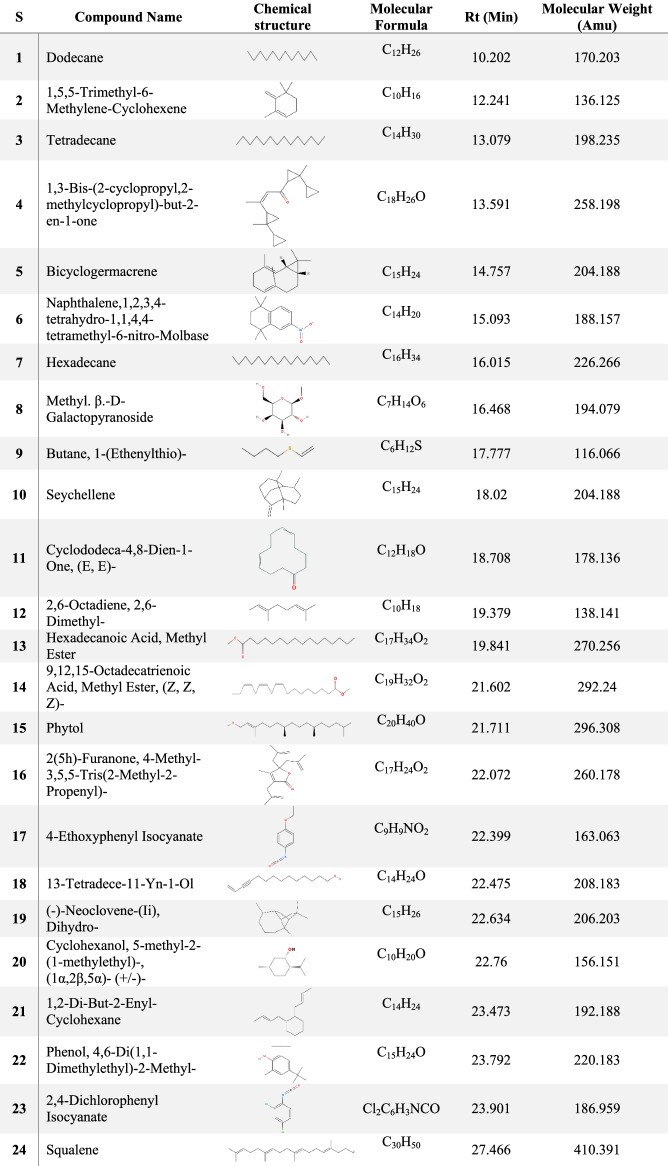
GC–MS analysis results of *Vitex agnus-castus* methanolic extract

**Table 3:**
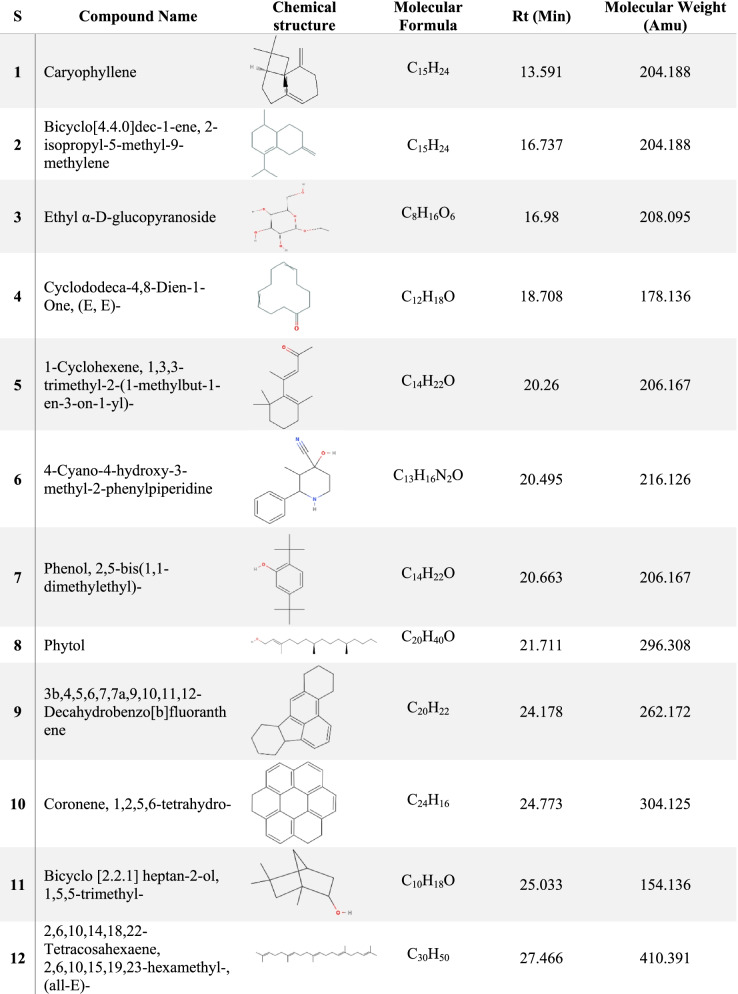
GC–MS analysis results of *Vitex agnus-castus* ethanolic extract

**Fig. 4:**
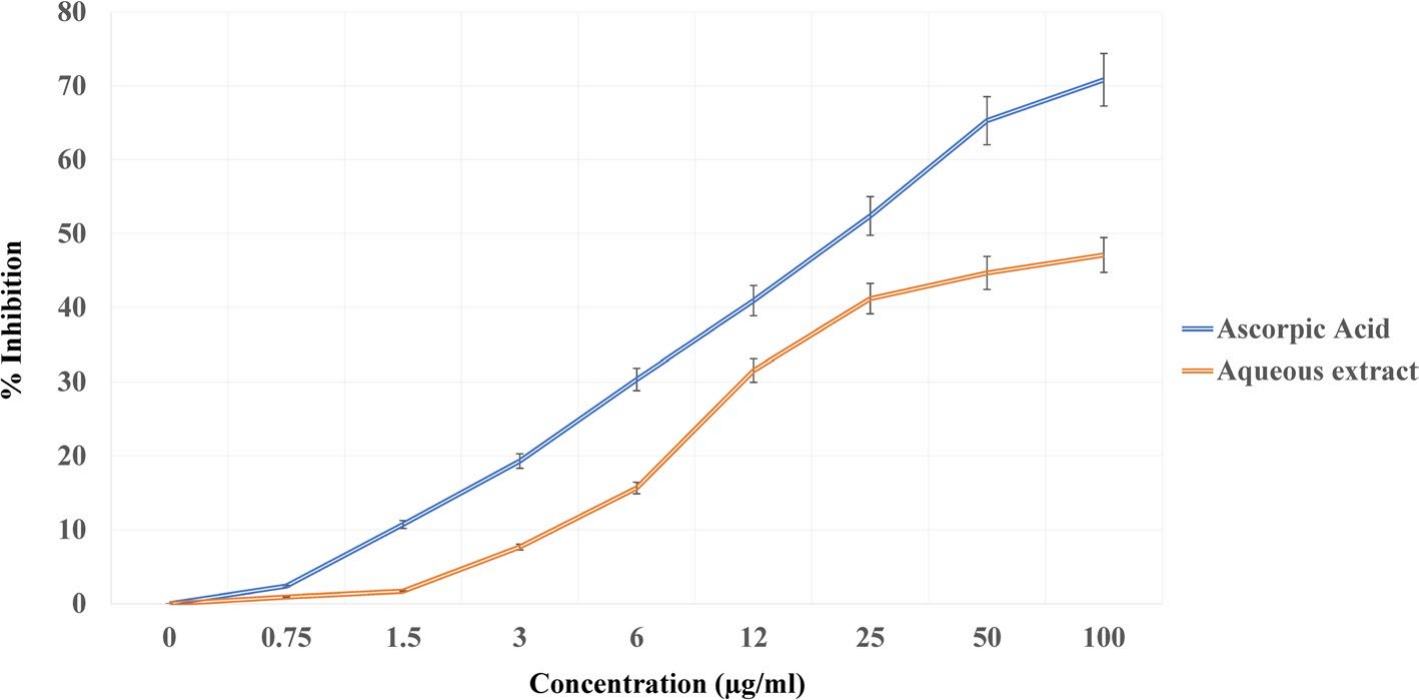
DPPH radical scavenging activity of *Vitex agnus-castus* leaves aqueous extract

**Fig. 5:**
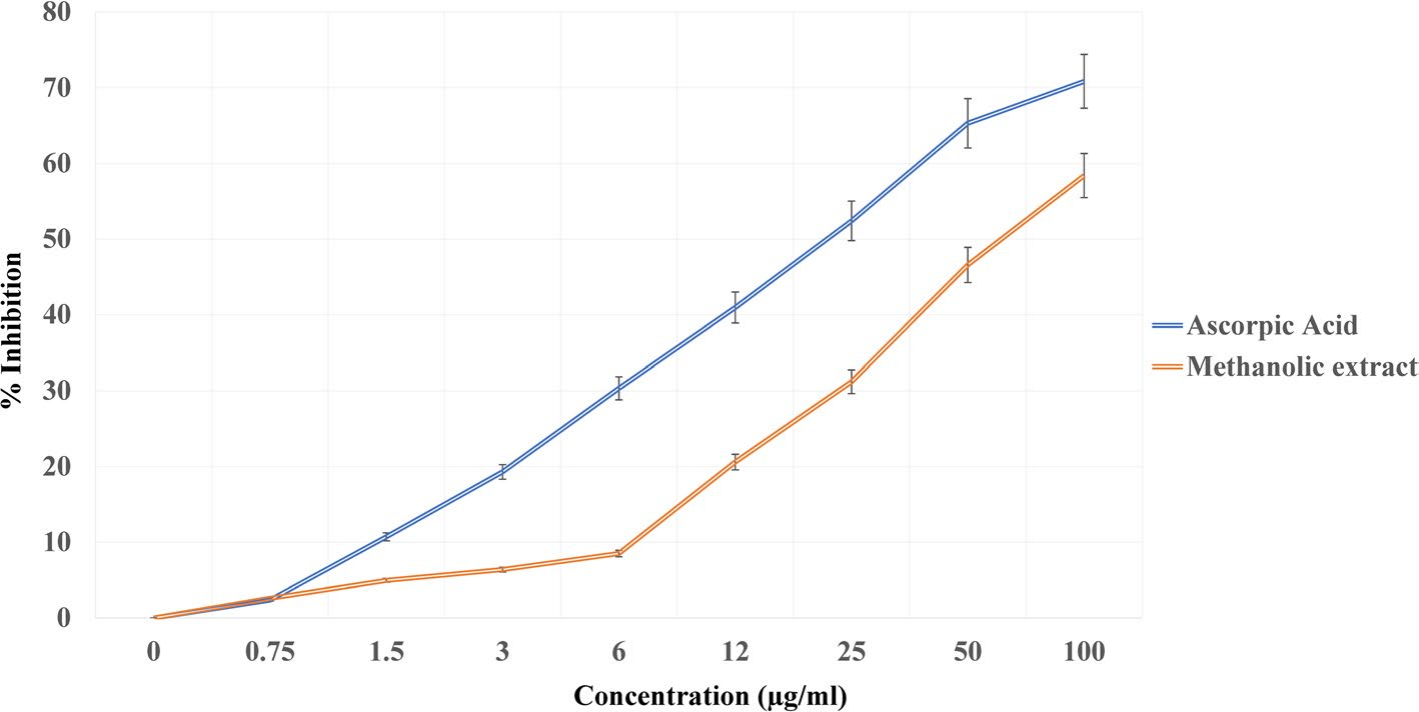
DPPH radical scavenging activity of *Vitex agnus-castus* leaves methanolic extract

**Fig. 6:**
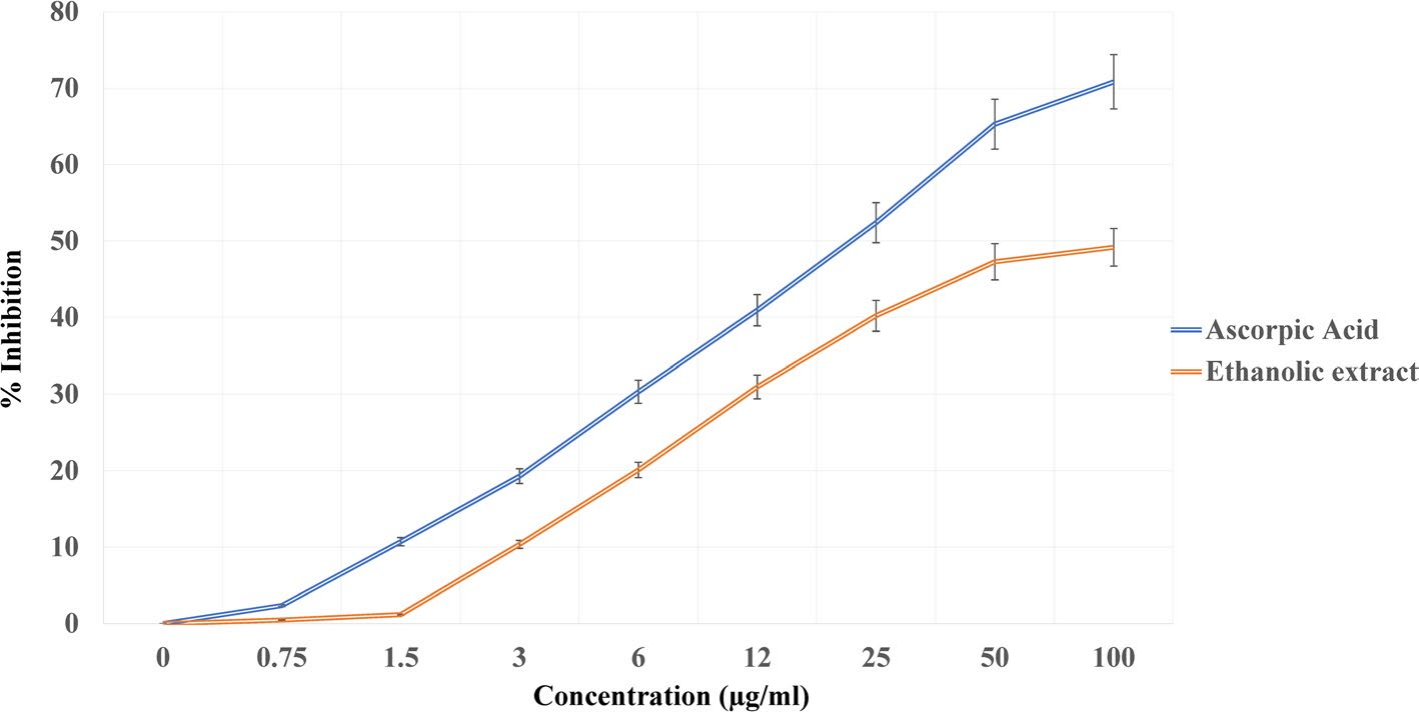
DPPH radical scavenging activity of *Vitex agnus-castus* leaves ethanolic extract

Antioxidants are extensively important substances that retain the capability to cover the body from damage caused by free revolutionary- convinced oxidative stress [26]. In the current study, the antioxidant activity for all *V. agnus castus* extracts was tested in the hunt for new bioactive composites from natural coffers (Figs. 4, 5, and 6). *V. agnus castus* aqueous, ethanol, and methanol extracts present the loftiest antioxidant exertion compared with reference antioxidant ascorbic acid, for DPPH scavenging exertion, attained results for DPPH agree with the phenol contents determined for each sample. The polyphenols content in different plant extracts acts as reducing agents and antioxidants by the hydrogen-giving property of their hydroxyl groups [40]. Hence, we could conclude that these polyphenols are responsible for the observed antioxidant exertion in this study.

### Different extracts from V. agnus-castus showed significant anti candida activity

**Fig. 7:**
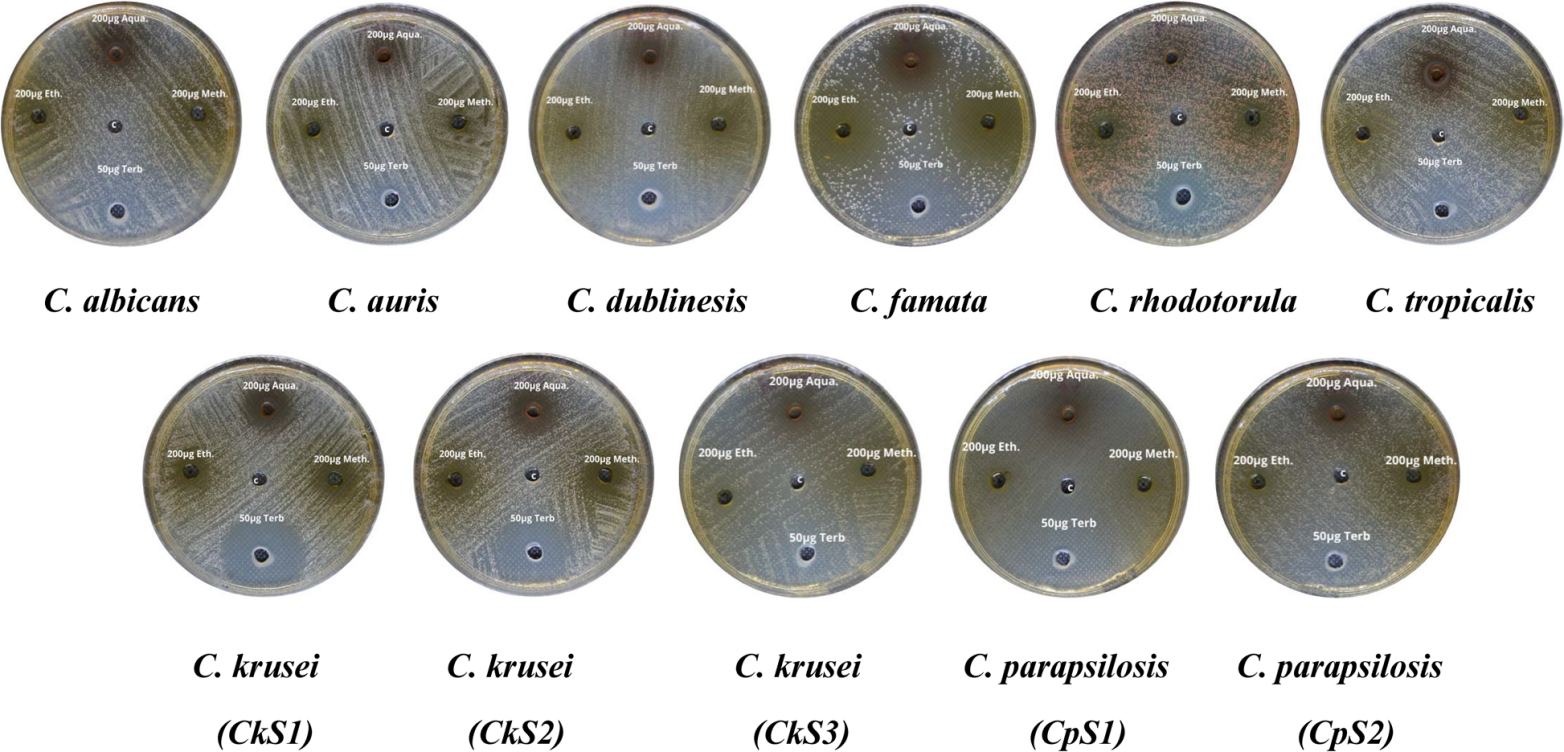
Antifungal effects of different *Vitex agnus-castus* leaves extracts against *Candida* species. Muller-Hinton Agar plates shoed the *Candida* growth inoculated with Aqueoues, Methanolic, and Ethanolic extracts *Vitex agnus-castus* extract, compared to Terbinafine antibiotic and control (c)

**Fig. 8:**
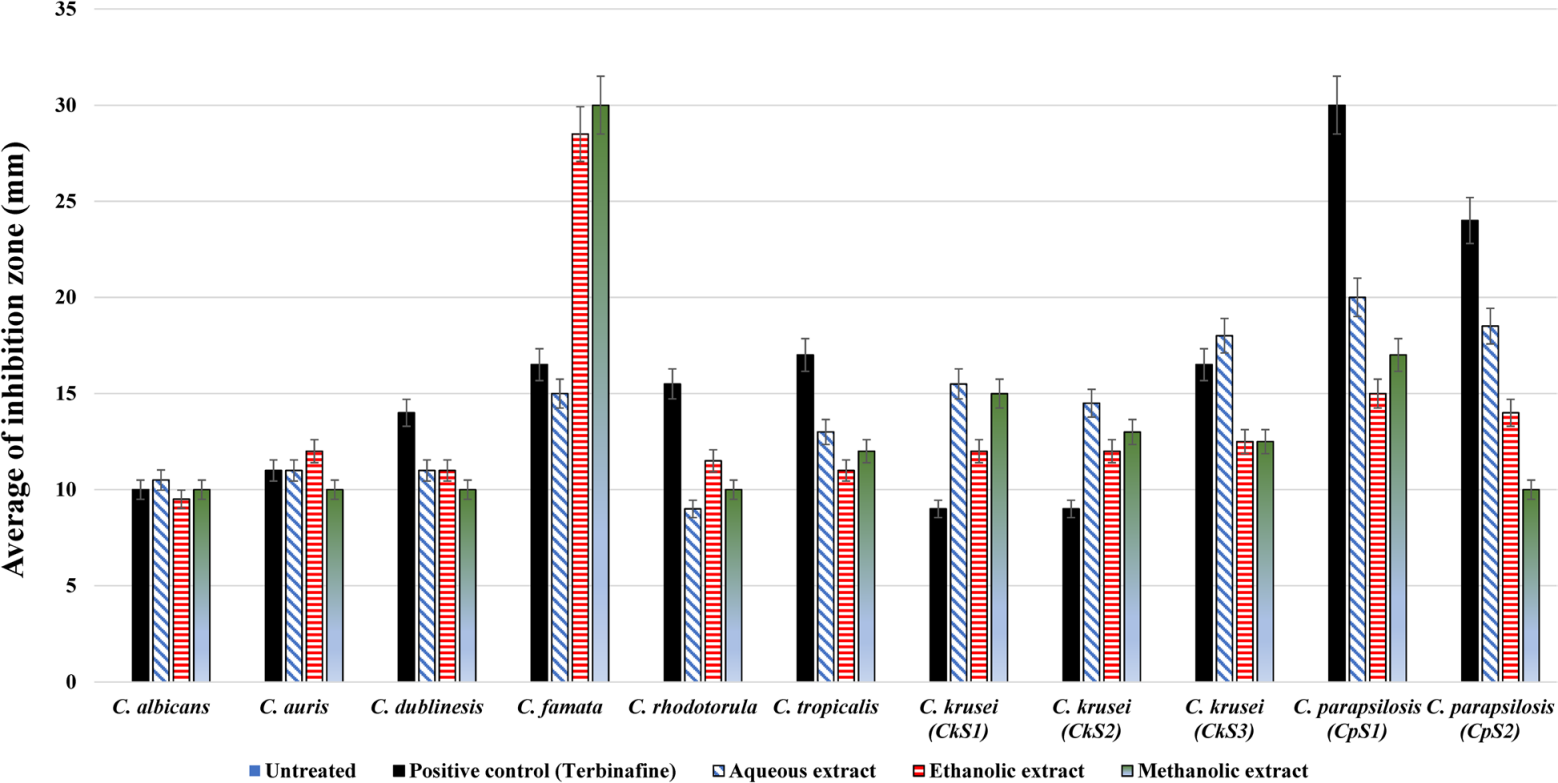
Antifungal effects of different *Vitex agnus-castus* leaves extracts against *Candida* species. Histogram chart of the anticandidal activities of aqueous, ethanolic and methanolic extracts, compared to Terbinafine antibiotic and control, by measuring the average of inhibition zone (mm)

Different concentrations of *V. agnus-castus* extracts were tested to evaluate their antifungal activity against different *Candida* sp. (Fig. 7). As shown in Table 4, the most effective concentration in all extracts was 200 µg/ml against all species, however, the 100 µg/ml of the aqueous extract showed inhibitory effects against *C. krusei* and *C. parapsilosis* different strains. Furthermore, the growth of *C. famata*, *C. parapsilosis (cp1), *and *C. parapsilosis (cp2)* were significantly affected by both 100 and 200 µg/ml concentrations of ethanolic extract (*P* = *0.003, 0.01, 0.04*) and methanolic extract (*P* = *0.0009, 0.02, 0.004*), respectively, as compared to the anticandidal positive control (Terbinafine). The aqueous extract induced significant growth inhibition of both *C. krusei (ck1)* (*P* = *0.03)* and *C. krusei (ck2) (P* = *0.03) *with the high dose of 200 µg/ml. Also, As shown in Fig. 8, aqueous extracts showed the highest inhibitory effect on *Candida* growth against all species except for *C. auris, C. famata, *and *C. rhodotorula. *The growth of *C. famata* was strongly affected by treatment with methanolic extract, where the ethanolic extract showed the largest zone of inhibition against *C. auris, C. dublinesis, *and *C. rhodotorula.* Compared to the inhibitory effects of the Terbinafine, the aqueous extract concentration of 200 µg/ml showed higher activity against *C. albicans*. Also, the ethanolic extract showed higher activity against *C. auris,* while both methanolic and ethanolic extracts showed inhibitory effects against *C. famata.* All extracts had the highest inhibitory effects against *C. krusei* species (Table 4, Fig. 8). MIC for *V. agnus-castus* extracts was calculated as 150 µg/ml for most species, except for *C. famata* in which the MIC was calculated at 75 µg/ml (Table 4). The aqueous extract was more potent for inhibiting Candida growth than both ethanolic and methanolic extracts.

**Table 4:** Screening of the minimal inhibitory concentration (MIC) of *Vitex agnus-castus* extract (µg/ml) by measuring the inhibition zone diameter (mm) for *Candida* strains

Strains	Aqueous extract				*p value*	MIC	Ethanolic extract				*p value*	MIC	Methanolic extract				*p value*	MIC	+ C
	**25**	**50**	**100**	**200**			**25**	**50**	**100**	**200**			**25**	**50**	**100**	**200**			
***C. albicans***	**-**	**-**	-	10.5	0.87	150	**-**	**-**	-	9.5	0.87	150	**-**	**-**	-	10	1.00	150	10
***C. auris***	**-**	**-**	-	11	1.00	150	**-**	**-**	-	12	0.76	150	**-**	**-**	-	10	0.76	150	11
***C. dublinesis***	**-**	**-**	-	11	0.42	150	**-**	**-**	-	11	0.42	150	**-**	**-**	-	10	0.29	150	14
***C. famata***	**-**	**-**	14	15	0.71	75	**-**	**-**	23.5	28.5	0.003*	75	**-**	**-**	26.5	30	0.0009*	75	16.5
***C. rhodotorula***	**-**	**-**	-	9	0.10	150	**-**	**-**	-	11.5	0.31	150	**-**	**-**	-	10	0.16	150	15.5
***C. tropicalis***	**-**	**-**	-	13	0.33	150	**-**	**-**	-	11	0.15	150	**-**	**-**	-	12	0.23	150	17
***C. krusei (ck1)***	**-**	**-**	14	15.5	0.03*	75	**-**	**-**	-	12	0.32	150	**-**	**-**	-	15	0.04*	150	9
***C. krusei (ck2)***	**-**	**-**	13	14.5	0.04*	75	**-**	**-**	-	12	0.32	150	**-**	**-**	-	13	0.18	150	9
***C. krusei (ck3)***	**-**	**-**	15	18	0.71	75	**-**	**-**	-	12.5	0.32	150	**-**	**-**	-	12.5	0.32	150	16.5
***C. parapsilosis (cp1)***	**-**	**-**	18	20	0.07	75	**-**	**-**	-	15	0.01*	150	**-**	**-**	-	17	0.02*	150	30
***C. parapsilosis (cp2)***	**-**	**-**	16	18.5	0.26	75	**-**	**-**	-	14	0.04*	150	**-**	**-**	-	10	0.004*	150	24

(-); no inhibition was noticed. + C; Terbinafine

^*^P value was calculated by Chi square test against + C

In contrast to our findings, a previous study showed that the ethanolic and methanolic extracts of *V. agnus-castus* L. leaves induced higher antifungal effects than the aqueous extract against *Pseudomonas aeruginosa* with MIC ranging from 1.56–25 µg/ml [41]. Another study showed that the concentration of 200 µg/ml of *V. agnus-castus* leaf different extracts had significant anticandidal activity against *C. tropicalis, C. albicans,* and *C. ciferrii, *however, the measured zones of inhibition were smaller than our results [38].

Multiple studies used different components extracted from natural plants to reduce the drug resistance observed against different pathogenic microbes. A previous study showed that the content of the essential oil extracted from *V. agnus-castus* L. seeds showed strong antimicrobial activity against *C. albicans, C. dubliniensis, C. glabrata, C. krusei, C. parapsilosis, C. lusitaniae, C. famata, *and *C. tropicalis *with a MIC of 130–213 µg/ml [42]. In the same manner, another study revealed that essential oil of *Ruta graveolens* affected the growth of *C. tropicalis and C. albicans* at MIC of 4.1 and 8.2 µg/mL, respectively, and that reduced the azole resistant [43]. On the cellular level, the cytocidal effects of essential oils extracted from *Austroeupatorium inulaefolium* revealed strong antimicrobial activity against resistant strains of *Candida* and bacteria at low concentrations [44]. Reportedly, other plant extracts possess antioxidant and antimicrobial properties due to the presence of phenolic compounds, such as flavonoids, hydroxybenzoic acids, and hydroxyphenyl propene [42]. Hence, *V. agnus-castus* might be used as a possible source of natural antifungal drugs.

**Fig. 9:**
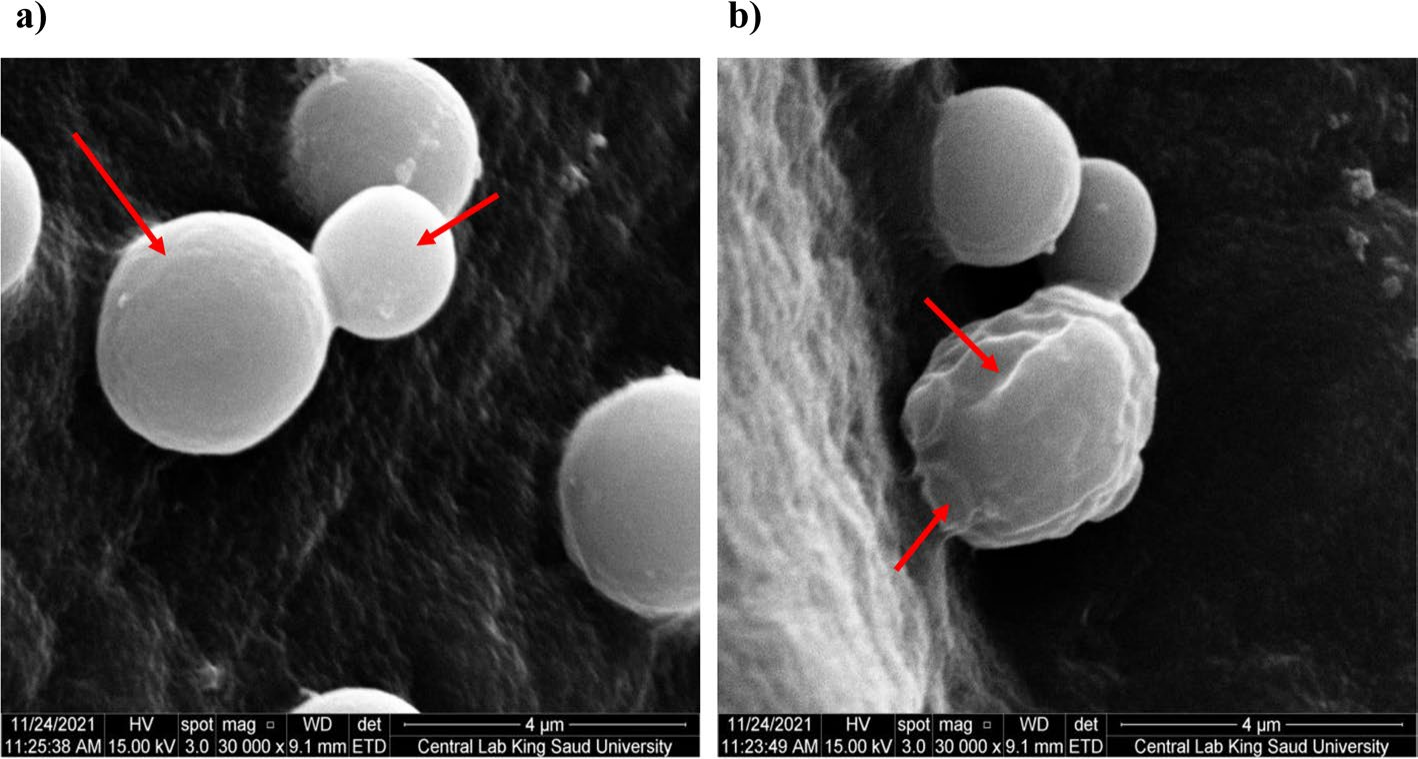
SEM imaging of *C. famata* showing the morphological changes with different treatments. *C. famata* cells were either a) untreated (Control) or b) treated with methanolic extract) treated with methanolic extract. Arrows indicated some morphological differences

To investigate the morphological and ultrastructure changes of the tested species, induced by *V. agnus-castus, *SEM analysis was performed against *C*. *famata* whose growth was the highest inhibited against the untreated control sample (Fig. 9). The control sample exhibited clear and well-demarcated cells that appeared with smooth walls and a defined bud morphology. The sample treated with methanolic extract showed misshapen, distorted, and damaged cell structures, revealed a swelling and rough appearance of the cell, and multiple blisters and bubbles formed on their surface (Fig. 9b**).** The microphotograph of *C. famata* showed distorted cells were flattened and had lost their normal morphology after treatment with the methanolic extract**.** The poor inhibitory activity of extracts against some *Candida* species, including *C. albicans* might be due to their inability to penetrate the cell membrane; some species of *Candida* synthesize biofilms [45]. The complex extracellular matrix of biofilm is mostly impermeable and may limit the penetration of antifungal drugs by binding to the antifungal agent and blocking target sites [45–48]. However, a previous study showed that the rich content of geraniol and linalool in some plants had a significant reduction in the number of viable biofilm cells of *C. tropicalis* and complete inhibition after 48 h of exposure [46]. Also, it was reported that *V. agnus-castus* leaves had high content of caffeic by 0.277% [49], where the Caffeic Acid Phenethyl Ester was found to inhibit the growth of different candida strains by affecting the biofilm-forming and maturation abilities, which caused their death [48]. All these studies robust the anticandidal activity of *V. agnus-castus* against different drug-resistant strains.

**Fig. 10:**
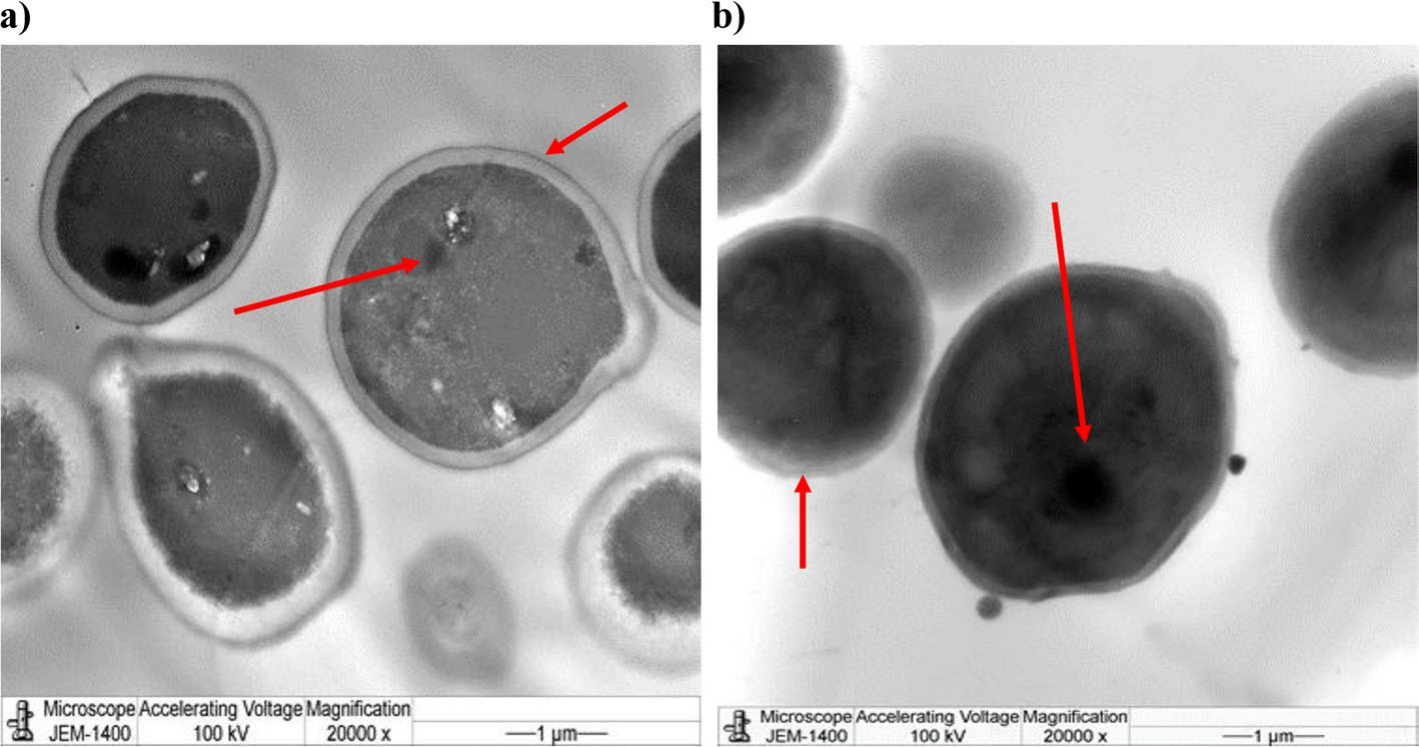
TEM imaging of *C. famata* showing the morphological changes with different treatments. *C. famata* cells were either a) untreated (Control) or b) treated with methanolic extract. Arrows indicate some morphological difference

The morphological changes seen in TEM were harmonious with SEM compliances. The microphotographs of cells, that didn't admit treatment, showed clear discrimination of cell membranes and walls, complete perimeters, and well-distributed cell organelles (Fig. 10a). Still, the treated cells (200 µg/ mL of methanolic excerpt) showed dramatic structural changes in organelles, accompanied by cell wall rupture in the utmost of the observed fields, dissolution or absence of cell membranes, contracted protoplasm, blebs, bulged cell membranes, distorted, and oohing cells. Some underdeveloped cells were also observed (Fig. 10b). The antifungal exertion of methanol extracts in this study might be due to a rich excerpt with numerous phytoconstituents. Methanol is an excellent detergent that excerpts numerous polar and many nonpolar composites; phenols, glycosides, coumarins, sesquiterpenes, and alkaloids may have antifungal exertion and are anticipated to be uprooted by methanol [24].

## Conclusion

Limited data is available about the medicinal uses of *V. agnus-castus*. More studies should be conducted to find alternative solutions, such as natural products, to control pathogens. The current study suggested that *V. agnus-castus* leaves extract might be used as an alternative anti-fungal treatment against *Candida* infections. Many phenolic and bioactive compounds were revealed, which might explain the observed anti-candida activities. More robust, randomized, controlled clinical trials would be desirable with well-characterized *V. agnus-castus* preparations to corroborate its beneficial effects in vivo.

## Data Availability

All data generated or analyzed during this study are included in this published article.
